# Stillbirth: Case definition and guidelines for data collection, analysis, and presentation of maternal immunization safety data^☆^

**DOI:** 10.1016/j.vaccine.2016.03.044

**Published:** 2016-12-01

**Authors:** Fernanda Tavares Da Silva, Bernard Gonik, Mark McMillan, Cheryl Keech, Stephanie Dellicour, Shraddha Bhange, Mihaela Tila, Diana M. Harper, Charles Woods, Alison Tse Kawai, Sonali Kochhar, Flor M. Munoz, The Brighton Collaboration Stillbirth Working Group

**Affiliations:** a GlaxoSmithKline Biologicals, Wavre, Belgium; b Wayne State University School of Medicine, Detroit, MI, USA; c The University of Adelaide, North Adelaide, South Australia, Australia; d PATH, Seattle, WA, USA; e Liverpool School of Tropical Medicine, Liverpool, United Kingdom; f Novartis Healthcare, Hyderabad, India; g Sanofi Pasteur, Lyon, France; h University of Louisville School of Medicine, Louisville, KY, USA; i Harvard Medical School and Harvard Pilgrim Health Care Institute, MA, USA; j Global Healthcare Consulting, India; k Baylor College of Medicine, Houston, TX, USA

**Keywords:** Stillbirth, Fetal death, Adverse event, Immunization, Guidelines, Case definition

## 1 Preamble

### 1.1 Need for developing case definitions and guidelines for data collection, analysis, and presentation for stillbirth as an adverse event following immunization during pregnancy

One of the most common adverse pregnancy outcomes is the death of the fetus. Fetal death has a great number of different and legally mandated definitions and particularly, different reporting requirements among different countries and states, which sometimes use different parameters, including birth weight, body length and/or the clinical estimate of gestational age thresholds [1]. Miscarriage (spontaneous abortion) and stillbirth are two general terms describing the death of the fetus, but they refer to losses that occur at different times during pregnancy. The distinction of these definitions affects the prospects for their accurate recording in vital registration systems or national stillbirth registries, community and hospital surveys, clinical research studies, together with those for measurements and comparisons. There is no universally accepted definition when a fetal death is called a stillbirth vs. spontaneous abortion; the reporting policies in the different countries and within the states of a same country are not uniformly followed and there are also differences in terms of how the gestational age is assessed and interpreted [1], [2], [3], [4].

The various definitions used therefore pose a methodological difficulty when attempting to interpret and accurately compare stillbirth rates and associated risk factors. It is therefore necessary to reach a consensus on the definition and classification for the adverse events in pregnancy data to be comparable as well as steps toward a more comprehensive evaluation of stillbirth.

Based on the WHO definition of third-trimester stillbirth used for international comparability, i.e. dead fetus of 1000 g or more at birth, or after 28 completed weeks of gestation, or attainment of at least 35 cm crown-heel length (see Table 1), at least 2.65 million cases of annual stillbirths were calculated worldwide in 2008, with 1.2 million of these fetal deaths occurring intrapartum [5], [6], [7].

The reported incidence of stillbirth varies significantly between studies from different countries and depending on the definitions used, but generally ranges from 3.1 to 6.2/1000 births or 1 in 160 deliveries [2], [8], [9]. The large majority of stillbirths (∼98%) occur in low/middle-income countries [1], [6], [7], [10], [11], [12]. With improvement in prenatal care, some of these deaths can be preventable. It is a fact that the overall incidence of stillbirth has declined overtime in developed countries by implementing appropriate healthcare policies for handling high-risk pregnant women. In low/middle-income countries, prevalence rates can be however inaccurate due to underreporting and documentation (e.g. home delivery) and reliable data are often difficult to obtain [10], [13], [14], [15], [16], [17].

#### 1.1.1 Causes and risk factors of stillbirth

The cause of the death of a fetus is often unknown, but can be attributable to various origins [2], [18], [19], [20], [21], [22], [23], [24], [25], [26]. It is important to recognize that there is a distinction between the underlying cause of the death (the disease process), the mode of death (for example asphyxia) and the classification of the death (e.g. growth restriction). Causes of stillbirth may also differ at different gestational ages.

A stillbirth of unknown cause is one that cannot be explained by any identifiable cause. The prevalence of stillbirths due to unknown causes varies from 25 to 60% of all fetal deaths, depending on the classification systems and evaluation of the deadborn fetus, e.g. the cause of death of the fetus who is small for gestational age can be attributed to the fetal growth restriction in some systems, but others consider it inexplicable if the underlying cause of the growth restriction is unknown [26], [27].The proportion of unclassified stillbirths can be significantly reduced with systems that use customized weight-for-gestational-age charts, such as the relevant condition at death (ReCoDe) system [22], or with systems that capture multiple and/or sequential contributing factors, such as Tulip, Perinatal Society of Australia and New Zealand – Perinatal Death Classification (PSANZ-PDC) or Causes Of Death and Associated Conditions (CODAC) [28]; moreover, stillbirth rates may differ when there is association with underlying determinants, for example, a lower risk of stillbirth is observed in a small for gestational age fetus if the mother is of short stature and has a multiple gestation [29].

Traditionally, the causes of stillbirth have been differentiated in maternal, fetal, placental and external factors. The most commonly quoted causes in the literature are as follows:

- Maternal causes: Maternal infection is one of the most important causes for stillbirth [20]. Common ascending infections (with or without membrane rupture) are due to *Escherichia coli*, *Klebsiella*, *Group B Streptococcus*, *Enterococcus*, *Mycoplasma*/*Ureaplasma*, *Haemophilus influenzae* and *Chlamydia* [30], [31]. In developing countries, other infectious agents can also be considered, e.g. malaria, syphilis and HIV [5]. One database cohort study conducted in England assessing viral infections as a cause of fetal loss in data from 1988 to 2008 concluded that more than one-third (37%) of the viral-attributed fetal deaths occurred antepartum, from parvovirus (63%) or cytomegalovirus (33%) [32]. Diabetes mellitus, thyroid abnormalities, hypertensive disorders, systemic lupus erythematosus, cholestasis of the pregnancy, renal disease, sickle-cell disease and other maternal medical conditions are also causes for stillbirth [2]. Anemia and nutritional deficiencies in the mother, common in low/middle-income countries, have been long debated to be also a cause of stillbirths or other adverse pregnancy outcomes [5]. In contrast, a high first hemoglobin measurement in early pregnancy has been shown to be associated with an almost 2-fold increase in risk of stillbirth [33].
- Fetal causes: Among these, poor fetal growth or intrauterine fetal growth restriction (IUGR) is considered one of the most frequent causes of stillbirth. Presumably, the growth restriction is due to a placental dysfunction which may be related to numerous maternal diseases or infections described above [34], [35], [36]. Other cited causes are: multiple gestation, congenital anomalies, genetic abnormalities, fetal infection, and post maturity [19], [20], [37], [38]. The most common genetic etiology for stillbirth is due to karyotype abnormalities, however many stillborn fetuses with normal karyotypes also have genetic abnormalities [39].
- Placental causes include placental abruption, premature rupture of membranes, vasa previa, chorioamnionitis, vascular malformations and umbilical cord accidents such as knots or abnormal placement [21], [40].
- External causes: Some common examples are: antepartum mother's injuries/trauma or delivery/labor incidents such as birth asphyxia and obstetric trauma. Where modern obstetric care is not available, deaths can be frequent. It is estimated that in developing countries asphyxia causes around seven deaths per 1000 births, whereas in developed countries this proportion is less than one death per 1000 births (5, 20). Availability of good delivery facilities also affects the pregnancy outcomes, as it was observed in a study that availability of skilled attendant during delivery (one of the factors in delivery process) lead to decline in stillbirth rate, however the authors concluded that this needs further analysis [41].

There are many known epidemiological risk factors for stillbirth. Systematic reviews have confirmed very early or advanced maternal age as risk factors. Moreover, nulliparous women have a higher risk of stillbirth than multiparous women across all ages. Of these, nulliparous women aged 35 years and older have been shown to have a 3.3-fold increase in the risk of unexplained fetal death compared with women younger than 35 years of age. The odds ratio for maternal age 40 years and older is 3.7 [42], [43].

Other factors associated with increased risk of stillbirth are: body mass index (BMI) ≥30, smoking (which includes active and passive smoking), substance abuse (especially cocaine, but also cannabis and alcohol), and multifetal gestation, with significantly higher rates of stillbirth observed in monochorionic twins than in dichorionic [2], [44], [45], [46], [47], [48]. One study showed that maternal overweight (i.e. Body Mass Index ≥25) increases the risk of antepartum stillbirth, especially term antepartum stillbirth, whereas weight gain per se during pregnancy was not associated with the risk of fetal death [49]. Women with a previous stillbirth are well known to be at 5- to 10-fold increased risk of recurrence for stillbirth. Also AB blood group appeared to be preferentially associated with stillbirth before 24 completed weeks of gestation [50].

Globally, black women have 2.2 fold increased risk of stillbirth compared to white women [51]. The black/white disparity in stillbirth hazard at 20–23 weeks is 2.75, decreasing to 1.57 at 39–40 weeks. Medical, pregnancy and labor complications account for 30% of the risk of stillbirth in Blacks and 20% in Whites and Hispanics. Trends have also show that stillbirth rates are slightly higher among male compared to female fetuses [51]. Worldwide, 67% of stillbirths occur in rural families, where skilled birth attendance and cesarean sections are much lower than that for urban births [52].

#### 1.1.2 Diagnosis of stillbirth

There are diverse existing methods/criteria for identifying stillbirths:

- Clinical signs: They are those that reflect absence of fetal vitality, either antepartum or by direct examination postpartum:
  a. Antepartum: mother does not feel fetal activity; the maternal weight is maintained or decreased, the fundal height stops increasing or even decreases if the reabsorption of amniotic fluid occurs. At the medical examination, intrauterine ascertainment of death is confirmed by the absence of fetal heart tones before delivery by auscultation methods (e.g. using Pinard horn, handheld Doppler, fetoscopy, doptone or stethoscope) or after electronic fetal heart monitoring/non-stress test. Auscultation of the fetal heart tones by Pinard horn, stethoscope or even handheld Doppler is insufficiently sensitive for a confirmatory diagnosis. In a series of 70 late pregnancies in which fetal heart tones were inaudible on auscultation, 22 were found to have viable fetuses [53]. Auscultation of fetal heart tones or misinterpreted experiences of fetal movements can also give false reassurance [54]; maternal pelvic blood flow can result in an apparently normal, but low, fetal heart rate pattern with handheld Doppler. The sign of Boero is the clear auscultation of maternal aortic beats due to the eventual absorption of amniotic fluid. The fetus becomes less perceptible to palpation as maceration progresses. The sign of Negri is the crackling or crepitation of the fetal head during its palpation. Sometimes vaginal dark blood loss is noted, there might be increased consistency of cervix because of the hormonal decline and also, appearance of secretion of colostrum in the mammary glands, although these signs are not specific.
  b. Postpartum ascertainment of death is confirmed by Apgar scores of 0 at 1 and 5 min, absence of vital signs including the documentation of no heart rate and respirations, absence of pulsation of the umbilical cord, and no definitive movement of voluntary muscles. Heartbeats are to be distinguished from transient cardiac contractions; respirations are to be distinguished from transient fleeting respiratory efforts or gasps. Macroscopic appearance of the fetus may show signs of maceration and the level of maceration can determine time of death. The earliest sign of macerations are seen in the skin 4–6 h after intrauterine death; desquamated skin measuring 1 cm or more in diameter and red or brown discoloration of the umbilical cord correlate with fetal death 6 or more hours before birth; desquamation involving the skin of face, back or abdomen with 12 or more hours; desquamation of 5% or more of the body surface with 18 or more hours; moderate to severe desquamation, brown skin discoloration of the abdomen with 24 or more hours and mummification is seen in fetuses who died 2 or more weeks before birth [55].
- Radiologic studies: In addition to the above clinical signs, other secondary features might be seen antepartum if eventually imaging techniques such as X-ray radiography are used: collapse of the fetal skull with overlapping bones due to liquefaction of the brain, hydrops, flattening of the cranial cavity, head asymmetry, fall of the mandible (sign of open mouth), or fetal bunching due to a loss of the normal curvature of the spine due to macerating spinal ligaments, which may appear completely collapsed resulting in unrecognizable fetal mass. In addition, there might be also intra-fetal gas within the heart, blood vessels and joints or a translucent peri-cranial halo due to accumulation of fluid in the subcutaneous tissue; when the image is complete gives double cranial halo called “holy crown” [56], [57], [58], [59], [60].
- Ultrasound (US): real-time ultrasonography is the gold standard for the accurate diagnosis of stillbirth antepartum. The advantage of this method lies in the precocity with which the diagnosis can be made, because real time ultrasound allows direct visualization of the fetal heart and the absence of cardiac activity, absence of aortic activity and the absence of movements of the body or limbs of the fetus (to be distinguished from periods of fetal physiological rest). Imaging can be technically difficult, particularly in the presence of maternal obesity, abdominal scars and oligohydramnios, but views can often be improved with new generation US or with color Doppler of the fetal heart and umbilical cord. Other secondary signs that can be seen at US are: the accumulation of fluid in the subcutaneous tissue (anasarca), pleural and peritoneal effusion, and the loss of the definition of fetal structures, which often reflect maceration.

#### 1.1.3 Stillbirth following immunization

Decades of vaccine use and evidence from clinical trial data and observational studies have shown the safety of traditional non-live vaccines (e.g. tetanus, pertussis or influenza) during pregnancy. Currently inactivated influenza virus, and pertussis vaccines are recommended for use during pregnancy in many parts of the world. Pertussis vaccines are generally available as part of combined vaccines such as tetanus toxoid, reduced diphtheria toxoid, and acellular pertussis (Tdap) vaccines, or Tdap with inactivated poliomyelitis virus vaccines (Tdap-IPV). Systematic reviews for inactivated influenza virus vaccines have concluded that the vaccine is not associated with an increased risk of stillbirth [61], [65], [67], [70]. One review paper describes that influenza vaccination might decrease the incidence of adverse outcomes of pregnancy such as stillbirth, as a result of the prevention of influenza infection related inflammation [61]. These findings were generalizable to monovalent influenza A (H1N1) vaccines, with the majority of evidence obtained for women immunized during their 2nd or 3rd trimester of pregnancy [61], [62], [63], [64], [65], [66], [67], [68], [69], [70], [71], [72], [73], [74], [75].

Fewer studies have examined stillbirth following Tdap administration during pregnancy, including two large retrospective studies completed in the US and the UK where stillbirth rates were compared to matched unvaccinated pregnant women and the authors concluded that the vaccine is not associated with an increased risk of stillbirth [76], [77], [78]. Remaining stillbirth data on pertussis containing vaccines comes from adverse event registries and small studies having similar findings [79], [80], [81]. Tetanus toxoid (TT) monovalent and tetanus toxoid reduced diphtheria (Td) vaccines are recommended for use in pregnancy in some countries where elimination of maternal and neonatal tetanus remains a priority [82].

Most live vaccines are contraindicated or not recommended for use during pregnancy [83]. Many of the live attenuated vaccines also come with a recommendation to avoid pregnancy for the month following immunization. This is due to the theoretical risk of transmission of the virus through the placenta to the fetus [82], [83]. Stillbirth data on many of these vaccines is derived from the follow up of women inadvertently immunized during early pregnancy. Rubella and varicella are of specific interest due to the potentially severe consequences of wild-type infection in susceptible pregnant women, which can lead to congenital rubella syndrome (CRS), and congenital varicella syndrome. Much of the research investigating the safety of the MMR and varicella vaccine has therefore looked at congenital anomalies outcomes. However, there is some data available on stillbirth rates following immunization showing no safety concerns [84], [85], [86]. A meta-analysis of eleven studies reported data on stillbirth (defined as fetal death ≥20 weeks of gestation) and found that the smallpox vaccination is not associated with an increased risk of stillbirth, pooled RR 1.03 (95% CI: 0.75–1.40) [87]. A study conducted in Finland during a mass oral poliovirus immunization campaign conducted between 1984 and 1986 reported stillbirth rates among women who were pregnant during the period of vaccination and whose infants were delivered at the three major hospitals in the Helsinki area between 0.4% and 0.6%, depending on their trimester of exposure, compared with 0.45% in the reference cohort [88].

### 1.2 Methods for the development of the case definition and guidelines for data collection, analysis, and presentation for stillbirth as an adverse events following immunization during pregnancy

Following the process described in the overview paper [89] as well as on the Brighton Collaboration Website http://www.brightoncollaboration.org/internet/en/index/process.html, the Brighton Collaboration Stillbirth Working Group was formed in 2015 and included members of clinical, academic, public health, research and industry background. The composition of the working and reference group as well as results of the web-based survey completed by the reference group with subsequent discussions in the working group can be viewed at: http://www.brightoncollaboration.org/internet/en/index/working_groups.html.

To guide the decision-making for the case definition and guidelines, a literature search was performed using Medline, Embase and the Cochrane Libraries, including the terms stillbirth, stillborn, intrauterine death, fetal demise, fetal mortality, fetal death, dead-born, fetal loss, intrapartum death, antepartum death, perinatal audit, perinatal death, perinatal mortality, pregnancy loss and vaccine, immunization and vaccination. Exhaustive search strategies were implemented using appropriate key words, accepted MeSH words, and combinations thereof. All abstracts were screened for possible reports of stillbirth following immunization. Searches were restricted to references in English, published since 1970 and involving only human subjects. Multiple general medical, pediatric, obstetrics and infectious disease text books were also searched.

The search and screening resulted in the identification of articles with potentially relevant material for further evaluation. This literature provided several different general definitions for stillbirth, its epidemiology, numerous descriptions for stillbirth causes and/or risk factors and the diagnostic criteria put forth. Most publications addressing stillbirth following immunization were case reports of single cases or case series describing various pregnancy outcomes, for which terminology was very inconsistent and very few used case definitions.

### 1.3 Rationale for selected decisions about the case definition of stillbirth as an adverse event following immunization during pregnancy

#### 1.3.1 The term stillbirth

In general, stillbirth is defined as a fetus with no signs of life prior to the complete expulsion or extraction from its mother, and after a pre-defined duration of gestation; after delivery, it is confirmed that the fetus does not show any evidence of life, and cannot be resuscitated.

The basic WHO definition for “stillbirth” is the intrauterine death of the fetus at any time during pregnancy [90]. However, for practical purposes, legal definitions usually require reportable fetal deaths to attain a gestational age (for stillbirth the GA generally considered is between 20 and 28 weeks) or a birth weight (generally between 350 and 1000 g). The minimum gestational age cut-off defining stillbirth vs. miscarriage generally varies from 20 to 28 weeks of gestation based on standards of fetal viability across countries, based on available medical care and health infrastructure [6]. In most high income and some middle income countries, thresholds vary from 18 to 22 weeks while in low income areas/countries thresholds are higher, up to 28 weeks [18]. The definition and ascertainment could be therefore different in developing/low-middle income vs. developed/high income countries. For international comparability, the WHO recommends using the cut-off of 1000 g or more at birth (if available), or after 28 completed weeks of gestation, or attainment of at least 35 cm crown-heel length [5]. In the United States, there are eight different definitions by combinations of gestational age and weight, and at least as many in Europe [91], [92].

In general, stillbirths are classified according to the gestational age, and are typically divided into early stillbirths (from 20 to 28 weeks gestation) and late stillbirths (after 28 weeks gestation). This division is based on those stillbirths that are difficult to prevent compared with those that are potentially preventable (i.e. late stillbirths). Stillbirths are also classified by whether death occurred before or after the onset of labor, referred as antepartum stillbirth and intrapartum stillbirth, respectively.

Despite all these sub classifications, the primary method for classification of stillbirth is according to the presumed cause [93]. In addition, there are over 35 classification systems to define stillbirth or perinatal death used in different countries around the world [18], [42], [94], [95], [96], [97], the most recent are the suggested ReCoDe [98], the modified Whitfield-Australia/New Zealand Classifications [99], and the World Health Organization's International Classification of Disease (ICD-10) systems [90] (see Table 1).

In this article, we will use the general term stillbirth, to refer to fetal deaths occurring after a pre-defined duration of gestation, in accordance with selected/preferred definitions used to fulfill the research needs in a given setting or to fit a reporting purpose, regardless of whether the death of the fetus could have occurred in utero (antepartum) or at the time of delivery (intrapartum).

The case definition presented in this document does not prescribe the use of a specific gestational age cut off or combination of gestational age and/or weight and size assessments to differentiate between miscarriage and stillbirth, but rather considers the currently utilized definitions of stillbirth worldwide and the importance of having a definition that is applicable in different clinical settings and environments. The variability in the definition of stillbirth stems from variability in viability cut offs in different settings, available resources, local practices, cultural influences, legal implications, and local and international reporting requirements. The WHO definitions take these elements in consideration and are widely used [5].

The working group emphasizes the importance of consistently and systematically capturing all cases of stillbirth in clinical trials assessing the safety of vaccines given during pregnancy. The study protocol should clearly describe the selected definition of a case of stillbirth and utilize it consistently throughout all study sites for data collection and analysis to ensure data comparability and a better understanding of this adverse pregnancy outcome. The working group recommends to make explicit a working definition of stillbirth to capture all events, for example “deadborn fetus at or after 22 completed weeks of gestation” and to consider categorization into other subgroups based on the goals of the study and relevant analyses, for example “early (after 22 weeks)” vs. “late (after 28 weeks)” stillbirth.

The working group suggests that differentiation of antepartum and intrapartum stillbirth is relevant, whenever possible, to understand potential underlying etiologies and mechanisms leading to the event. However, when this differentiation is not possible, the outcome will be recorded as a stillbirth, defined as the delivery of a fetus with no signs of life and assessed by the attendant and/or investigator to be within the gestational age consistent with the selected cut off in the definition.

#### 1.3.2 Related term(s) of stillbirth

There are different terms used within this context. Those terms are: stillborn, intrauterine death, fetal/fetal demise, fetal/fetal mortality, fetal/fetal death, dead-born and fetal/fetal loss. Other less specific terms are sometimes used as well: intrapartum death, antepartum death, perinatal audit, perinatal death, perinatal mortality, pregnancy loss.

#### 1.3.3 Formulating a case definition that reflects diagnostic certainty: weighing specificity vs. sensitivity

It needs to be re-emphasized that the grading of definition levels is entirely about diagnostic certainty, not clinical severity or causality of an event. Detailed information about the severity of the event should additionally always be recorded, as specified by the data collection guidelines.

The number of symptoms and/or signs that will be documented for each case may vary considerably. The case definition has been formulated such that the Level 1 definition is highly specific for the condition. As maximum specificity normally implies a loss of sensitivity, two additional diagnostic levels have been included in the definition, offering a stepwise increase of sensitivity from Level One to Level Three, while retaining an acceptable level of specificity at all levels. In this way it is hoped that all possible cases of stillbirth can be captured.

#### 1.3.4 Rationale for individual criteria or decision made related to the case definition

There is a need to consider data sources and availability of existing data when defining pregnancy outcomes in research. The interpretation of data is difficult when cut-off values of the definitions differ, and it is also problematic in multiple gestations with both live and dead siblings. Flexibility and alignment with existing definitions where studies/surveillance are performed are necessary to ensure comparability and interpretation of data. Another consideration for case inclusion criteria are deliveries that occur outside of the hospital setting (e.g. home delivery), in the absence of medical personnel, and then are presented to the hospital as a death. Sometimes these data are not made available. In addition, under these circumstances, it is not always possible to determine whether the fetus was stillborn, or if the fetus lived for any length of time.

Although very few data may be available to determine a cause of stillbirth, the assessment of the cause includes the macroscopic examination of the fetus for congenital malformations, and if available, autopsy and karyotype; cord and placental examination and pathology, documenting antepartum events such as maternal factors, fetal factors (e.g. intrauterine growth restriction), external factors (e.g. trauma), and peri-partum events such as preterm premature rupture of membranes (PPROM), infection, abruption, cord events, laboratory findings, etc. These data (i.e. pathology and laboratory findings) may not be included in the case definition of stillbirth, but are recommended to be obtained in the data analysis to ascertain the possible cause.

#### 1.3.5 Determination of the gestational age at death

The gestational age (GA) seems to be the most widely used criterion to define stillbirth. Several algorithms are available for assessment of gestational age at death based on available clinical data and simple examination of the infant after delivery [100]. These may be used when other means of determining gestational age are unavailable.

The most common method for the ascertainment of estimated Gestational Age (GA) at time of fetal death is based on the Last Menstrual Period (LMP): The duration of gestation is measured from the first day of the last normal menstrual period. Gestational age is expressed in weeks. Other methods include measurement of fundal height, biometric parameters of the fetus which can be determined antepartum by US or by other less accurate measurement methods post-partum, such as fetal crown-to-heel length or foot length [100], [101], or the direct observation of the fetal maturation, if no measurement methods are available. Different scoring systems are also used to estimate the gestational age after birth but all involve neurologic reflexes and/or physical characteristics such as skin and cartilage changes, however all these neurologic measures are not possible for stillbirths and skin and cartilage changes are unreliable if there is maceration.

A proposed algorithm for estimating GA for studies in various community settings is presented in a related manuscript (Preterm Birth Definition and GA assessment algorithm – available at http://www.brightoncollaboration.org). This algorithm presents criteria based on different parameters that could be available, including LMP and different measurement methods including ultrasound scan, or stillborn assessment immediately after birth. In obese women, or when uterine anatomy is otherwise compromised (e.g. multiple fibroids), clinician determination of GA by “best assessment” is to be used. Although GA is determined antepartum, findings must be consistent with immediate and simple examination of the stillborn fetus after delivery, otherwise a post hoc determination is needed. Assessment of gestational age of the fetus is a key component of the case definition of stillbirth. The working group recommends the use of the GA assessment algorithm in the “Preterm Birth” Brighton Collaboration Case Definition for the assessment of gestational age in the mother or fetus.

#### 1.3.6 Timing post immunization in pregnancy

We postulate that a definition designed to be a suitable tool for testing causal relationships requires ascertainment of the outcome (e.g. stillbirth) independent from the exposure (e.g. immunizations).

Further, stillbirth often occurs outside the controlled setting of a clinical trial or hospital. In some settings it may be impossible to obtain a clear timeline of the event, particularly in less developed or rural settings and in the observational research setting via retrospective medical record reviews. In order to avoid selecting against such cases, the Brighton Collaboration case definition avoids setting arbitrary time frames. An exact time-frame should not be offered since it would have to refer to a wide range of signs and symptoms without a scientific evidence base. Using an arbitrarily restrictive set point might bias future data collection unnecessarily. Therefore, to avoid selection bias, a restrictive time interval from immunization to onset of stillbirth should not be an integral part of such a definition, but is recommended to be used in the data analysis to examine factors such as temporal clusters. Where feasible, details of this interval should be assessed and reported as described in the data collection guidelines (see guideline 34, section 3.2).

### 1.4 Guidelines for data collection, analysis and presentation

As mentioned in the overview paper, the case definition is accompanied by guidelines which are structured according to the steps of conducting a clinical trial, i.e. data collection, analysis and presentation. Neither case definition nor guidelines are intended to guide or establish criteria for management of ill infants, children, or adults. Both were developed to improve data comparability.

### 1.5 Periodic review

Similar to all Brighton Collaboration case definitions and guidelines, review of the definition with its guidelines is planned on a regular basis (i.e. every three to five years) or more often if needed.

## 2 Case definition of stillbirth^2^

### 2.1 Stillbirth

Is a fetal death occurring before birth after a selected, pre-defined duration of gestation (see Table 1). The death of the fetus could have occurred before the onset of labor^3^ (antepartum) or at the time of delivery (intrapartum). For all levels of diagnostic certainty, the definition of stillbirth must include:

- Determination of absence of signs of life^4^ in the fetus or newborn
  **AND**
- Determination of fetal/newborn gestational age through maternal information or through fetal/newborn evaluation (see Preterm Birth Definition – Assessment of Gestational Age)

#### 2.1.1 Antepartum stillbirth

Antepartum stillbirth is defined as fetal death occurring during pregnancy and prior to delivery, before the onset of labor. It is usually diagnosed prior to delivery, but may not be diagnosed until after the infant is delivered. The infant is born without signs of life.^3^

#### 2.1.2 Intrapartum stillbirth

Intrapartum stillbirth is defined as fetal death occurring after the onset of labor and prior to delivery. The infant is born without signs of life.^3^ Documentation of a live fetus prior to or at the onset of labor exists.

*Additional findings that might be helpful to differentiate between Antepartum and Intrapartum Stillbirth at the time of delivery*:

• Physical Examination: Fetuses who died antepartum can have skin changes consistent with maceration, tissue injury, meconium staining, and edema.
• Laboratory/pathology: Autopsy examination of the fetus and/or the placenta.

### 2.2 Stillbirth ascertainment of levels of certainty

#### 2.2.1 Antepartum Stillbirth

Fetal death occurs prior to the evidence of labor.

**Level 1**
  • Delivery of an infant with no of signs of life at birth (No spontaneous movements, no umbilical cord pulse, no heartbeat, no respirations, Apgar score of 0 at 1 and 5 min) determined by physical examination after delivery (with or without electronic monitoring of heart rate, respiratory rate, and pulse oximetry).
    **AND**
  • Prenatal ultrasound examination documenting lack of fetal cardiac activity or movement before the onset of labor.
    **OR**
  • Auscultation for fetal heart tones (using electronic devices or non-electronic devices) documenting lack of fetal heartbeat.
    **AND**
  • Maternal report of lack of fetal movement for 24 h or more.
    **OR**
  • Maternal physical examination confirming lack of fetal movement.
    **OR**
  • Radiology findings consistent with intrauterine fetal death.
    **AND**
  • Attended delivery followed by fetal physical examination after birth consistent with antepartum death, by obstetrician, neonatologist, pediatrician, maternal-fetal medicine specialist, or pathologist. In the setting where access to a specialist is not feasible, diagnosis by a health care provider trained or experienced to make the diagnosis is acceptable (e.g. general practice physician, mid-wife, nurse practitioner, a physician's assistant or other qualified trained practitioner).
    **OR**
  • Fetal/placental pathology report consistent with antepartum death.
    **AND**
  • Gestational age within pre-defined range for selected stillbirth definition as assessed by maternal and/or fetal parameters (Level 1 or 2 in GA assessment algorithm).
**Level 2**
  • Delivery of an infant with no of signs of life at birth (No spontaneous movements, no umbilical cord pulse, no heartbeat, no respirations, Apgar score of 0 at 1 and 5 min) determined physical examination after delivery.
    **AND**
  • Maternal report of lack of fetal movement for 24 h or more.
    **OR**
  • Maternal physical examination confirming lack of fetal movement.
    **OR**
  • Auscultation for fetal heart tones (using electronic or non-electronic devices) documenting lack of fetal heartbeat.
    **AND**
  • Attended delivery followed by physical examination after birth consistent with antepartum death, by specialist or qualified trained practitioner appropriate to the health care setting.
    **OR**
  • Fetal/placental pathology report consistent with antepartum death.
    **AND**
  • Gestational age within pre-defined range for selected stillbirth definition as assessed by maternal and/or fetal parameters (Level 1–2 in GA assessment algorithm).
**Level 3**
  • Delivery of an infant reported to have no of signs of life at birth (No spontaneous movements, no umbilical cord pulse, no heartbeat, no cry or spontaneous respirations, no chest movement, and whole body cyanosis).
    **AND**
  • Maternal report of lack of fetal movement for 24 h or more prior to delivery.
    **OR**
  • Report of auscultation for fetal heart tones (using electronic or non-electronic devices) documenting lack of fetal heartbeat.
    **AND**
  • Non-attended delivery followed by physical examination of the fetus after birth consistent with antepartum death by a health care professional appropriate to the level of standard of care in the health care setting.
    **OR**
  • Verbal history by a trained health care provider, non-medical witness or the mother of a fetus born with no signs of life or unresponsive to resuscitation efforts immediately after birth and with physical features consistent with antepartum death.
    **AND**
  • Gestational age within pre-defined range for selected stillbirth definition as assessed by maternal and/or fetal parameters (Level 2–3 in GA assessment algorithm).
**Level 4**
  • Report of stillbirth but fetus is not available for physical examination after birth (no objective assessment can be made).
  • Maternal information insufficient to assess gestational age.

#### 2.2.2 Intrapartum stillbirth

Fetal death occurs during labor and before delivery

**Level 1**
  • Delivery of an infant with no of signs of life at birth, including: No spontaneous movements, no umbilical cord pulse, no heartbeat, no respirations, and Apgar score of 0 at 1 and 5 min.
  • Determination of the absence of signs of life is made by physical examination after delivery, with or without electronic monitoring of heart rate, respiratory rate, and pulse oximetry.
    **AND**
  • Evidence of live fetus prior to onset of labor (documentation of fetal movement and of fetal heart tones by ultrasound prior to onset of labor) (*Note*: in the absence of evidence of a live fetus prior to the onset of labor, the fetal death should be reported as a stillbirth or an antepartum stillbirth).
    **AND**
  • Attended delivery followed by physical examination after birth consistent with intrapartum death by obstetrician, neonatologist, pediatrician, maternal-fetal medicine specialist, pathologist. In the setting where access to a specialist is not feasible, diagnosis by a health care provider trained or experienced to make the diagnosis is acceptable (e.g. general practice physician, mid-wife, or other qualified trained practitioner).
    **AND**
  • Gestational age within pre-defined range for selected stillbirth definition as assessed by maternal and/or fetal-neonatal parameters (Level 1 in GA assessment algorithm)
**Level 2**
  • Delivery of an infant with no of signs of life at birth, including: No spontaneous movements, no umbilical cord pulse, no heartbeat, no respirations, and Apgar score of 0 at 1 and 5 min.
  • Determination of the absence of signs of life is made by physical examination after delivery, with or without electronic monitoring of heart rate, respiratory rate, and pulse oximetry OR documentation of lack of response to resuscitation efforts.
    **AND**
  • Evidence of live fetus prior to onset of labor (maternal report of fetal movement prior to onset of labor and documentation of fetal heart tones by auscultation or hand held Doppler) (*Note*: in the absence of evidence of a live fetus prior to the onset of labor, the fetal death should be reported as a stillbirth or an antepartum stillbirth).
    **AND**
  • Attended delivery followed by physical examination after birth consistent with intrapartum death by a health care professional appropriate to the level of standard of care in the health care setting.
    **AND**
  • Gestational age within pre-defined range for selected stillbirth definition as assessed by maternal and/or fetal parameters (Level 1–2 in GA assessment algorithm).
**Level 3**
  • Delivery of an infant reported to have no of signs of life at birth, including: No spontaneous movements, no umbilical cord pulse, no heartbeat, no cry, no spontaneous respirations or chest movement, and whole body cyanosis.
    **AND**
  • Evidence of live fetus prior to onset of labor (maternal report of fetal movement prior to onset of labor OR auscultation of fetal heart tones) (*Note*: in the absence of evidence of a live fetus prior to the onset of labor, the fetal death should be reported as a stillbirth or an antepartum stillbirth).
    **AND**
  • Non-attended delivery followed by physical examination of the fetus after birth consistent with intrapartum death by a health care professional appropriate to the level of standard of care in the health care setting OR verbal history by a trained health care provider, non-medical witness or the mother of a fetus born with no signs of life or unresponsive to resuscitation efforts immediately after birth.
    **AND**
  • Gestational age within pre-defined range for selected stillbirth definition as assessed by maternal and/or fetal parameters (Level 2–3 in GA assessment algorithm).
**Level 4**
  • Report of stillbirth but fetus is not available for physical examination after birth (no objective assessment can be made).
  • Maternal information insufficient to assess gestational age.

## 3 Guidelines for data collection, analysis and presentation of stillbirth

It was the consensus of the Brighton Collaboration Stillbirth Working Group to recommend the following guidelines to enable meaningful and standardized collection, analysis, and presentation of information about stillbirth. However, implementation of all guidelines might not be possible in all settings. The availability of information may vary depending upon resources, geographical region, and whether the source of information is a prospective clinical trial, a post-marketing surveillance or epidemiological study, or an individual report of stillbirth. Also, these guidelines have been developed by this working group for guidance only, and are not to be considered a mandatory requirement for data collection, analysis, or presentation.

### 3.1 Data collection

These guidelines represent a desirable standard for the collection of available pregnancy outcome data following immunization to allow comparability. The guidelines are not intended to guide the primary reporting of stillbirths to a surveillance system. Investigators developing a data collection tool based on these data collection guidelines also need to refer to the criteria in the case definition.

Guidelines 1–43 below have been developed to address data elements for the collection of adverse event information as specified in general drug safety guidelines by the International Conference on Harmonization of Technical Requirements for Registration of Pharmaceuticals for Human Use [107], and the form for reporting of drug adverse events by the Council for International Organizations of Medical Sciences [108]. These data elements include an identifiable reporter and patient, one or more prior immunizations, and a detailed description of the adverse event, in this case, of stillbirth following immunization. The additional guidelines have been developed as guidance for the collection of additional information to allow for a more comprehensive understanding of stillbirth following immunization.

#### 3.1.1 Source of information/reporter

For all cases and/or all study participants, as appropriate, the following information should be recorded:

(1) Date of report.
(2) Name and contact information of person reporting^5^ and/or diagnosing the stillbirth as specified by country-specific data protection law.
(3) Name and contact information of the investigator responsible for the subject, as applicable.
(4) Relation to the patient (e.g. immunizer [clinician, nurse], family member [indicate relationship], other).

#### 3.1.2 Vaccinee/control

##### 3.1.2.1 Demographics

For all cases and/or all study participants (i.e. pregnant women and newborn), as appropriate, the following information should be recorded:

(5) Case/study participant identifiers (e.g. participant's first name initial followed by last name initial) or code (or in accordance with country-specific data protection laws).
(6) Participant's age at enrolment, race/ethnicity and gestational age at the time of enrolment.
(7) For dead newborn: Gestational age and birth weight/height.

##### 3.1.2.2 Clinical and immunization history

For all cases and/or all study participants, as appropriate, the following information should be recorded:

(8) Past medical history, including hospitalizations, underlying diseases/disorders, pre-immunization signs and symptoms including identification of indicators for, or the absence of, a history of allergy to vaccines, vaccine components or medications; food allergy; allergic rhinitis; eczema; asthma.
(9) Any medication history (including treatment for the event described) prior to, during, and after immunization including prescription and non-prescription medication as well as medication or treatment with long half-life or long term effect (e.g. immunoglobulins, blood transfusion and immune-suppressants) or substance abuse (e.g. narcotics or other recreational drug, alcohol or smoking).
(10) Immunization history (i.e. previous immunizations and any adverse event following immunization (AEFI), in particular occurrence of stillbirth after a previous immunization.
(11) Medical confirmation of live fetus prior to maternal immunization.

#### 3.1.3 Details of the immunization

For all cases and/or all study participants, as appropriate, the following information should be recorded:

(12) Date and time of maternal immunization(s).
(13) Description of vaccine(s) (name of vaccine, manufacturer, lot number, dose (e.g. 0.25 mL, 0.5 mL, multi-dose vial, etc.), number of dose if part of a series of immunizations against the same disease and vaccine diluent if separate from the vaccine container itself).
(14) The anatomical sites (including left or right side) of all immunizations (e.g. vaccine A in proximal left lateral thigh, vaccine B in left deltoid).
(15) Route and method of administration (e.g. intramuscular, intradermal, subcutaneous, and needle-free (including type and size), other injection devices).
(16) Needle length and gauge.
(17) Gestational age of the pregnancy at the time of immunization

#### 3.1.4 The adverse event

(18) For all cases at any level of diagnostic certainty and for reported events with insufficient evidence, the criteria fulfilled to meet the case definition should be recorded.
  Specifically document (if available):
(19) Clinical description of signs and symptoms of stillbirth, and if there was medical confirmation of the event (i.e. patient seen by physician).
(20) Date/time of onset,^6^ first observation^7^ and diagnosis^8^; as well as end of episode^9^ and final outcome,^10^ if appropriate (e.g. if the event no longer meets the case definition of stillbirth at the lowest level of the definition). For an event that meets the case definition of stillbirth, the end of episode is the same as date/time of onset, and the outcome is fatal (i.e. it results in death of the fetus).
(21) Concurrent signs, symptoms, and diseases.
(22) Pregnancy, labor and delivery details:
  • Pregnancy details: date of last normal menstrual period, ultrasound examinations, antenatal care visits, pregnancy-related illnesses and complications.
  • Labor and delivery details: for intrapartum fetal death specifically document (if available) mode of delivery and complications (e.g. fetal distress, antepartum/postpartum hemorrhage, assisted delivery, etc.).
(23) Measurement/testing
  • Values and units of routinely measured parameters (e.g. temperature, blood pressure) – in particular those indicating the severity of the event;
  • Method of measurement (e.g. type of thermometer, oral or other route, duration of measurement, etc.);
  • Results of laboratory examinations, surgical and/or pathological findings and diagnoses if present.
(24) Treatment given for stillbirth, especially specify what medications and dosing, as well as other interventions.
(25) Outcome^9^ at last observation (e.g. for an event that meets the case definition of stillbirth, it results in death of the fetus). Add descriptions if antepartum/intrapartum or postpartum maternal death occurred. Also, for multiple gestation, if concomitant twin death occurred.
(26) Objective clinical evidence supporting classification of the event as “serious”^11^ (i.e. results in death of the fetus).
(27) Exposures other than the immunization before and after immunization (e.g. food, environmental) considered potentially relevant to the reported event.

#### 3.1.5 Miscellaneous/general

(28) The duration of follow-up reported during the surveillance period should be predefined likewise (in this case, birth or delivery). It should aim to continue to resolution of the event (i.e. the outcome of the pregnancy is captured).
(29) Methods of data collection should be consistent within and between study groups, if applicable.
(30) Follow-up of cases should attempt to verify and complete the information collected as outlined in data collection guidelines 1–27.
(31) Investigators of patients with stillbirth should provide guidance to reporters to optimize the quality and completeness of information provided.
(32) Reports of Stillbirth should be collected throughout the study period regardless of the time elapsed between immunization and the adverse event. If this is not feasible due to the study design, the study periods during which safety data are being collected should be clearly defined.

### 3.2 Data analysis

The following guidelines represent a desirable standard for analysis of data on Stillbirth to allow for comparability of data, and are recommended as an addition to data analyzed for the specific study question and setting.

(33) Reported events should be classified in one of the following five categories including the three levels of diagnostic certainty. Events that meet the case definition should be classified according to the levels of diagnostic certainty as specified in the case definition. Events that do not meet the case definition should be classified in the additional categories for analysis.
  **Event classification in 5 categories**^12^
  • Event meets case definition
    (1) Level 1: Criteria as specified in the Stillbirth case definition
    (2) Level 2: Criteria as specified in the Stillbirth case definition
    (3) Level 3: Criteria as specified in the Stillbirth case definition
  • Event does not meet case definition
    *Additional categories for analysis*
    (4) Reported stillbirth with insufficient evidence to meet the case definition^13^
    (5) Not a case of stillbirth^14^
(34) The interval between immunization and reported stillbirth could be defined as the date/time of immunization (last vaccination) to the date/time of onset^8^ of the event, consistent with the definition. If few cases are reported, the concrete time course could be analyzed for each; for a large number of cases, data can be analyzed in the following increments for identification of temporal clusters:

**Table:** **Subjects with Stillbirth by Interval to Presentation**.

Interval*	Number (Percentage)
≤24 h after immunization	
2–≤7 days after immunization	
8–≤42 days after immunization	
>42 days after immunization	
Weekly unit increments thereafter	
Total
(35) If more than one measurement of a particular criterion is taken and recorded, the value corresponding to the greatest magnitude of the adverse experience could be used as the basis for analysis. Analysis may also include other characteristics like qualitative patterns of criteria defining the event.
(36) The distribution of data (as numerator and denominator data) could be analyzed in predefined increments (e.g. measured values, times), where applicable. Increments specified above should be used. When only a small number of cases is presented, the respective values or time course can be presented individually.
(37) Data on stillbirth obtained from subjects receiving a vaccine should be compared with those obtained from an appropriately selected and documented control group(s) and whenever possible with background rates of the event in non-exposed populations. Data should be analyzed by study arm and dose where possible, e.g. in prospective clinical trials.

### 3.3 Data presentation

These guidelines represent a desirable standard for the presentation and publication of data on stillbirth following immunization to allow for comparability of data, and are recommended as an addition to data presented for the specific study question and setting. Additionally, it is recommended to refer to existing general guidelines for the presentation and publication of randomized controlled trials, systematic reviews, and meta-analyses of observational studies in epidemiology (e.g. statements of Consolidated Standards of Reporting Trials (CONSORT), of Improving the quality of reports of meta-analyses of randomized controlled trials (QUORUM), and of Meta-analysis Of Observational Studies in Epidemiology (MOOSE), respectively) [109], [110], [111].

(38) All reported events of stillbirth should be presented according to the categories listed in guideline 33.
(39) Data on possible stillbirth events should be presented in accordance with data collection guidelines 1–32 and data analysis guidelines 33–37.
(40) Data should be presented with numerator and denominator (n/N) (and not only in percentages), if available.Although immunization safety surveillance systems denominator data are usually not readily available, attempts should be made to identify approximate denominators. The source of the denominator data should be reported and calculations of estimates be described (e.g. manufacturer data like total doses distributed, reporting through Ministry of Health, coverage/population based data, etc.).
  (41) The incidence of cases in the study population should be presented and clearly identified as such in the text.
  (42) If the distribution of data is skewed, median and inter-quartile range are usually the more appropriate statistical descriptors than a mean. However, the mean and standard deviation should also be provided.
  (43) Any publication of data on stillbirth should include a detailed description of the methods used for data collection and analysis as possible. It is essential to specify:
    • The study design;
    • The method, frequency and duration of monitoring for stillbirth;
    • The trial profile, indicating participant flow during a study including drop-outs and withdrawals to indicate the size and nature of the respective groups under investigation;
    • The type of surveillance (e.g. passive or active surveillance);
    • The characteristics of the surveillance system (e.g. population served, mode of report solicitation);
    • The search strategy in surveillance databases;
    • Comparison group(s), if used for analysis;
    • The instrument of data collection (e.g. standardized questionnaire, diary card, report form);
    • Whether the day of immunization was considered “day one” or “day zero” in the analysis;
    • Whether the date of onset^8^ and/or the date of first observation^9^ and/or the date of diagnosis^10^ was used for analysis; and
    • Use of this case definition for stillbirth, in the abstract or methods section of a publication.^15^

## Figures and Tables

**Table 1:** Existing conventional definitions for Stillbirth.

Source	Gestational Age (weeks)	Birth weight (g)	Height criteria (crown-heel length)	Definition
USA (CDC)	≥20 0/7	≥350		The US federal guidelines recommend reporting those fetal deaths whose birth weight is of 350 g or more, or if weight is unknown, of 20 completed weeks gestation or more, calculated from the date last normal menstrual period; the death shall be reported within 5 days after delivery to the Office of Vital Statistics or as otherwise directed by the State Registrar. Forty-one areas use a definition very similar to the federal definition, thirteen areas use a shortened definition of fetal death, and three areas have no formal definition of fetal death. Only 11 areas specifically use the term ‘stillbirth’, often synonymously with late fetal death; however they are split between whether stillbirths are irrespective of the duration of pregnancy, and whether some age or weight constraint is applied [92].
WHO/ICD (use for general statistics and registration)	≥22 0/7	≥500	≥25	The International Classification of Diseases, 10th revision (ICD-10) defines a fetal death as: “*death prior to the complete expulsion or extraction from its mother of a product of conception, irrespective of the duration of pregnancy; the death is indicated by the fact that after such separation the fetus does not breathe or show any other evidence of life, such as beating of the heart, pulsation of the umbilical cord, or definite movement of voluntary muscles without specification of the duration of pregnancy*”. WHO/ICD defines stillbirths as the death of a fetus that has reached a birth weight of 500 g, or if birth weight is unavailable, gestational age of 22 weeks or crown-to-heel length of 25 cm. Within this category, ICD classifies late fetal deaths (greater than 1000 g or after 28 weeks) and early fetal deaths (500–1000 g or 22–28 weeks). The legal requirements for registration of fetal deaths vary between and even within countries. WHO recommends that, if possible, all fetuses and infants weighing at least 500 g at birth, whether alive or dead, should be included in the statistics. The inclusion in national statistics of fetuses and infants weighing between 500 g and 1000 g is recommended both because of its inherent value and because it improves the coverage of reporting at 1000 g and over [5], [7].
WHO/ICD (for International comparison and reporting)	≥28 0/7	≥1000	≥35	The WHO recommends using the higher limit (1000 g/28 weeks/35 cm) of third-trimester stillbirths for international comparisons and reporting [5], [7].
EMA	≥22 0/7			The European Medicines Agency (EMA) uses the term stillbirth as the synonym of late fetal death, which is the death after 22 completed weeks of gestation [102]
NICHD – SCRN US, VPDC Australia	≥20 0/7	≥400		The Stillbirth Collaborative Research Network (SCRN) defines stillbirth as Fetal death at ≥20 completed weeks of gestation or ≥400 g birth weight. In Australia, stillbirth is also defined as fetal death (no signs of life), whether antepartum or intrapartum, at ≥20 weeks of gestation or ≥400 g birthweight, if gestational age is unknown, and it must be registered [103], [104].
ACOG (US)	≥20 0/7	≥350		The American College of Obstetricians and Gynecologists (ACOG) defines stillbirth as delivery of fetus which shows no signs of life e.g. absence of breathing, heart beats, pulsations in umbilical cord are absent, no voluntary movement of muscle. The suggested requirement is to report fetal deaths at 20 weeks or greater of gestation (if the gestational age is known) or a weight greater than or equal to 350 g if the gestational age is not known. The cut-off of 350 g is the 50th percentile for weight at 20 weeks gestation [2].
UK	≥24 0/7			The United Kingdom defines stillbirth as fetal death at 24 or more completed weeks of gestation [105], [106].

## Footnotes

2 The case definition should be applied when there is no clear alternative diagnosis for the reported event to account for the combination of symptoms.

3 The onset of labor is defined as regular, painful uterine contractions resulting in progressive cervical effacement and dilatation.

4 Signs of life include: spontaneous movements, spontaneous respirations, and spontaneous cardiac activity.

5 If the reporting center is different from the vaccinating center, appropriate and timely communication of the adverse event should occur.

6 The date and/or time of onset is defined as the time post immunization, when the first sign or symptom indicative for stillbirth occurred. This may only be possible to determine in retrospect.

7 The date and/or time of first observation of the first sign or symptom indicative for stillbirth can be used if date/time of onset is not known.

8 The date of diagnosis of an episode is the day post immunization when the event met the case definition at any level.

9 The end of an episode is defined as the time the event no longer meets the case definition at the lowest level of the definition.

10 Example: recovery to pre-immunization health status, spontaneous resolution, therapeutic intervention, persistence of the event, sequelae, death.

11 An AEFI is defined as serious by international standards if it meets one or more of the following criteria: (1) it results in death, (2) is life-threatening, (3) it requires inpatient hospitalization or results in prolongation of existing hospitalization, (4) results in persistent or significant disability/incapacity, (5) is a congenital anomaly/birth defect, (6) is a medically important event or reaction. For stillbirth, the event meets the definition of serious (i.e. it results in death of the fetus).

12 To determine the appropriate category, the user should first establish, whether a reported event meets the criteria for the lowest applicable level of diagnostic certainty, e.g. Level three. If the lowest applicable level of diagnostic certainty of the definition is met, and there is evidence that the criteria of the next higher level of diagnostic certainty are met, the event should be classified in the next category. This approach should be continued until the highest level of diagnostic certainty for a given event could be determined. Major criteria can be used to satisfy the requirement of minor criteria. If the lowest level of the case definition is not met, it should be ruled out that any of the higher levels of diagnostic certainty are met and the event should be classified in additional categories four or five.

13 If the evidence available for an event is insufficient because information is missing, such an event should be categorized as “Reported stillbirth with insufficient evidence to meet the case definition”.

14 An event does not meet the case definition if investigation reveals a negative finding of a necessary criterion (necessary condition) for diagnosis. Such an event should be rejected and classified as “Not a case of stillbirth”.

15 Use of this document should preferably be referenced by referring to the respective link on the Brighton Collaboration website (http://www.brightoncollaboration.org).
