# Supplementary material for: Stillbirth: Case definition and guidelines for data collection, analysis, and presentation of maternal immunization safety data
Source: Vaccine. 2016 Dec 1;34(49):6057–68. doi: 10.1016/j.vaccine.2016.03.044 (PMC5139804; doi:10.1016/j.vaccine.2016.03.044)
Supplement: Supplementary file 1 [file mmc1.docx]

**APPENDIX A: Tool to aid in classification of stillbirth vs. abortion/miscarriage and live birth with or without neonatal death**

**Pregnancy Outcomes**

**Neonatal death preterm**

< 37 weeks gestation

**Neonatal death at term**

> 37 weeks gestation

**Antepartum Stillbirth**

**Intrapartum Stillbirth**

**Live Birth**

**+/-**

**Neonatal Death**

**Live birth preterm**

< 37 weeks gestation

**Live birth at term**

> 37 weeks gestation

**Stillbirth**

Fetal death at

> 20 weeks gestation or

> 22 weeks gestation or

> 24 weeks gestation or

> 28 weeks gestation

**Abortion / Miscarriage**

Fetal death at

< 20 weeks gestation or

< 22 weeks gestation or

< 24 weeks gestation or

< 28 weeks gestation
